# Degradation Kinetics of Atorvastatin under Stress Conditions and Chemical Analysis by HPLC

**DOI:** 10.3390/molecules18021447

**Published:** 2013-01-24

**Authors:** Marcelo Antonio Oliveira, Maria Irene Yoshida, Valdenir José Belinelo, Romanélia Spessemille Valotto

**Affiliations:** 1 University Center of the North of Espirito Santo, UFES, BR 101 North, km 60, 29932-540 São Mateus, ES, Brazil; 2 Department of Chemistry, Federal University of Minas Gerais, Av. Pres. Antônio Carlos, 6627-31270-901 Belo Horizonte, MG, Brazil

**Keywords:** atorvastatin, intrinsic stability, degradation kinetics

## Abstract

Atorvastatin is an antilipemic drug belonging to the statins class, whose reference drug is Pfizer’s Lipitor^®^. It is used to reduce the levels of lipoproteins rich in cholesterol and reduce the risk of coronary artery disease. It is well-known that calcium atorvastatin (ATV), C_66_H_68_CaF_2_N_4_O_10_•3H_2_O, presents polymorphism. The drug in question is commonly sought after by pharmaceutical industries that produce generic drugs, due to the fact that the drug has a high value price, it is consumed globally, and its patent expired in late 2010. Many questions concerning this drug’s pharmaceutical scope demonstrate its importance regarding stability studies and the identification of degradation products of drugs and pharmaceutical formulations. ATV has been found to degrade under acid and basic conditions, including a first order kinetic degradation under acid conditions, as compared to a zero order kinetic degradation under basic conditions, which tends to be less stable when studied within acid mediums. The rate constant (k) for degradation in acid medium was 1.88 × 10^−2^ s^−1^ (first order), while for basic medium k = 2.35 × 10^−4^ mol L^−1^ s^−1^ (zero order), demonstrating a lower stability of the drug within acid mediums.

## 1. Introduction

Lipitor^®^, the commercial brand name of atorvastatin, was the best selling drug in the World from 2002 to 2009, generating a gross revenue of approximately 9.3 billion dollars. The patent protection granted by the pipeline was extended by court ruling until 28 December 2010 [1].

Atorvastatin calcium (ATV) is used to reduce the levels of lipoproteins rich in cholesterol and reduce the risk of coronary artery disease. This is due to this drug’s inhibitory action on the hydroxymethylglutaryl-CoA reductase (HMG-CoA reductase) enzyme, which is important in cholesterol biosynthesis [2].

**Figure 1:**
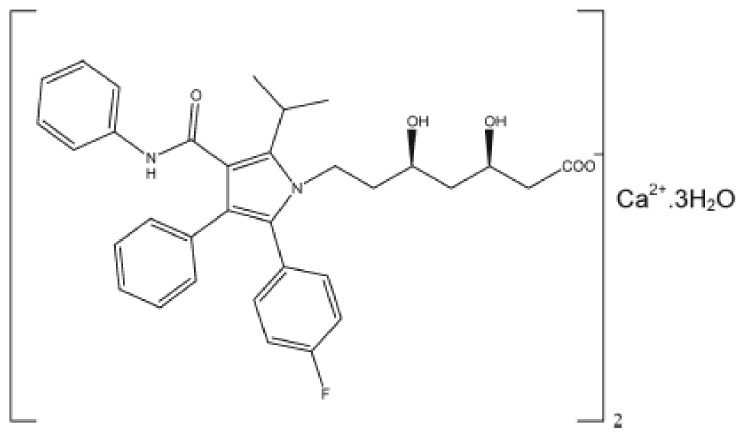
ATV (I).

ATV (I, Figure 1) presents a molecular formula of C_66_H_68_CaF_2_N_4_O_10_•3H_2_O and a molecular weight of 1,209.4 g/mol. ATV is a white crystalline powder with a partition coefficient [log P (octanol/water)] of 6.36 and a dissociation constant (pKa) of 4.46, presenting a fusion between 159.2 °C and 160.7 °C. The drug is insoluble in aqueous solutions with pH ≤ 4.0; very slightly soluble in water, phosphate buffer (pH 7.4) and acetonitrile; slightly soluble in ethanol; and very soluble in methanol [3,4]. It is well-known that the drug presents polymorphisms [5,6].

In the development of a pharmaceutical formulation in addition to a identifying polymorphism, it is important to determine the intrinsic stability of the drug to predict possible reactions and degradation products [7,8]. The intrinsic stability of the substance should be evaluated in terms of temperature, humidity, oxidation, UV light exposure, and hydrolysis at different pH values [7,8]. The photostability test can be evaluated under the conditions recommended by ICH Q1B [9], by subjecting the substance to ultraviolet irradiation. Some degradation pathways can be complex; however, not all decomposition products formed under conditions of intrinsic, yet more drastic, stability can be observed in the drug when subjected to the official conditions of the stability studies [7,10,11,12].

Stability studies and degradation kinetics are a integral parts of the quality control of a drug or medicinal product on an industrial scale. Degradation kinetics is also used to evaluate the stability under certain conditions as well as to compare stress conditions. Therefore, the intrinsic stability and kinetic studies are fundamental elements in the search for possible degradation products of drugs; however, these products do not commonly appear under normal drug storage conditions.

## 2. Results and Discussion

### 2.1. Validation of Analytical Method and Research of Degradation Products

The HPLC/UV-DAD method was validated for atorvastatin, which included: a capacity factor (k’) of 1.34; a peak symmetry (As) of 1.2; a theoretical plates/column (N) of 11,554; repeatability and intermediate precision (RSD less than 2%); intra-day and inter-day accuracies with a percentage recovery of 94.19% and 94.44%. The linear correlation coefficient (r) proved to be greater than 0.99 when in the range from 14 to 26 μg/mL. A detection limit of 0.45 μg/mL and a quantification limit of 1.36 μg/mL were applied. Selectivity studies, performed after drug stress conditions, and robustness proved to be appropriate.

**Figure 2:**
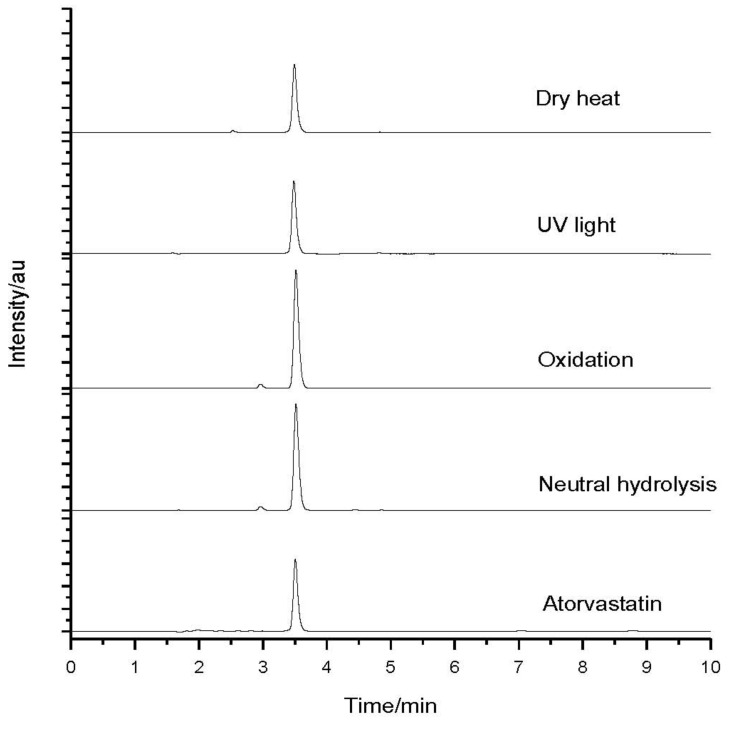
Atorvastatin chromatograms before (bottom) and after stress conditions: neutral hydrolysis; oxidation; exposure to UV light; and exposure to temperature (dry heat).

**Figure 3:**
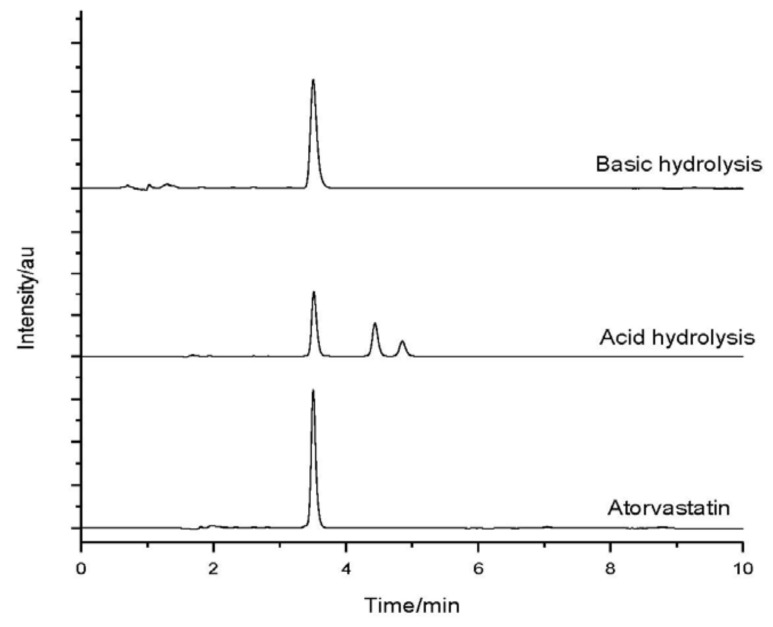
Atorvastatin chromatograms before (bottom) and after stress conditions: acid hydrolysis; and basic hydrolysis.

After having been submitted to stress conditions, the samples were analyzed by HPLC. Figure 2 shows the chromatograms of the drug and the samples subjected to stress when exposed to dry heat, UV light exposure, oxidation, and neutral hydrolysis. Figure 3 shows the chromatograms of the drug and the samples subjected to stress under acid and basic hydrolyses.

**Figure 4:**
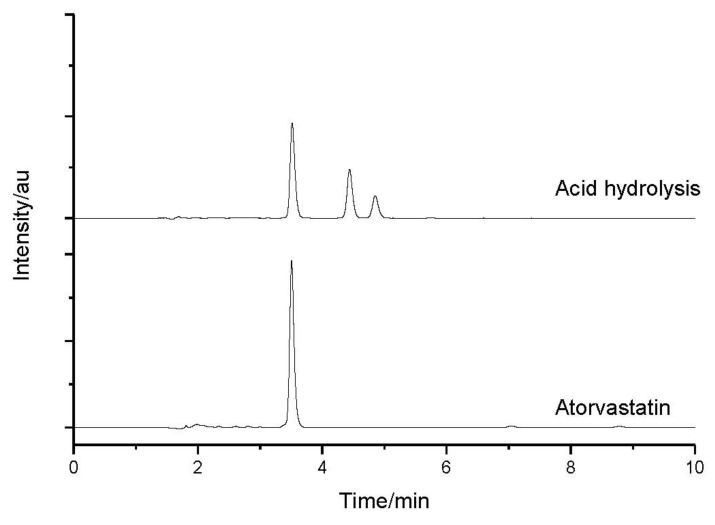
Atorvastatin chromatograms before (bottom) and after stress conditions in acid hydrolysis.

The ATV produced a retention time (t_R_) of 3.517 and degraded under acid and basic conditions. It could also be observed that the peak for t_R_ (retention time) of 3.0 min, as regards in the sample subjected to oxidation, represents the peak of hydrogen peroxide. The sample submitted to an acid medium showed a partial degradation with the formation of two degradation products, with t_R_ = 4.440 and t_R_ = 4.853, as seen in Figure 4.

**Figure 5:**
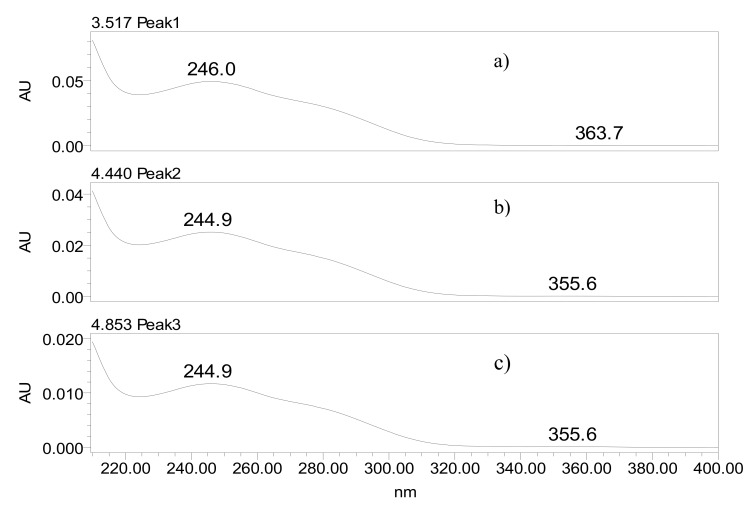
Spectra obtained from detector UV/DAD: (**a**) drug (t_R_ = 3.517), (**b**) degradation product 1 (t_R_ = 4.440), (**c**) degradation product 2 (t_R_ = 4.853).

Using the UV/DAD detector made it possible to trace the UV spectra concerning the peaks of the drug (t_R_ = 3.517), the degradation product 1 (DP1; t_R_ = 4.440), and the degradation product 2 (DP2; t_R_ = 4.853), as seen in Figure 5. The chromatographic resolutions (Rs) between the peaks were of 5.89 for ATV and DP1, and 2.39 to DP1 and DP2.

The graphs illustrate that the behavior of the UV spectra of degradation products is similar to the spectrum of the drug with the same λ_max_, indicating that the structure of the chromophore remains the same in the degradation products. In the sample subjected to basic hydrolysis, the degradation was observed by the reduction in the peak area of the drug.

### 2.2. The Degradation Kinetics under Acid and Basic Conditions

**Figure 6:**
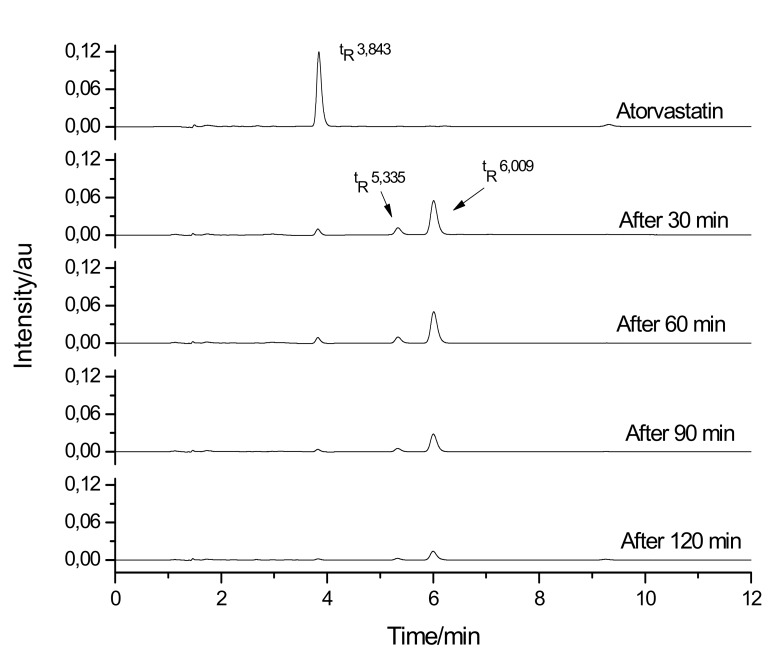
Chromatograms for atorvastatin degradation in acid medium.

Under both acid and basic conditions, where degradation of the drug occurred, the kinetics of degradation fitted mathematical models of zero, first, and second orders. After having been submitted to the stress conditions in an acid medium, a reduction in the drug concentration (t_R_ = 3.843 min), according to the time of exposure to stress, could also be observed. In addition, a consequent increase in the concentration of degradation products, DP1 (t_R_ = 5.335 min) and DP2 (t_R_ = 6.009 min), could be observed (Figure 6 and Table 1).

Considering the results in Table 1, it was possible to obtain the degradation kinetics of the drug under acid conditions for models of the zero, first, and second orders (Table 2).

Within the acid medium, the first order represented the kinetics of best fit, which presented a linear correlation coefficient (r) of close to 1.0000. According to this model, the rate constant (k) for the drug was 1.88 × 10^−2^ s^−1^.

**Figure 7:**
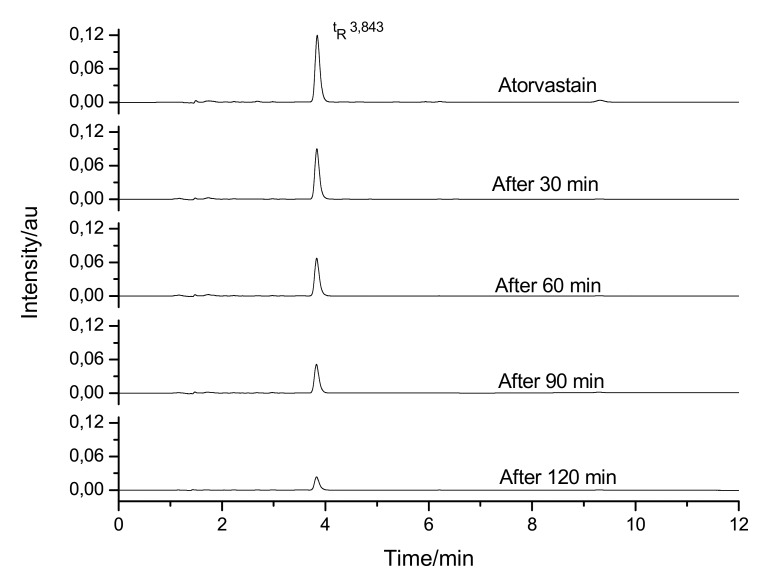
Chromatograms for atorvastatin degradation in basic medium.

ATV was also subjected to basic hydrolysis, and degradation can be seen in the graphs (Figure 7).

According to the results, it could be observed that the drug undergoes hydrolytic degradation when in the basic medium. However, despite the degradation, it was impossible to identify a degradation product. Considering the data of the chromatogram, it could be observed that the drug degraded as the peak diminished, as can be seen in Table 3.

With the results in Table 3, it was possible to obtain the degradation kinetics of the drug under basic conditions for zero, first and second order models, as seen in Table 4.

In the basic medium, the zero order represented the kinetics of best fit, presenting a linear correlation coefficient (r) of close to 1.0000. The rate constant (k) for the drug was 2.35 × 10^−4^ mol L^−1^ s^−1^.

## 3. Experimental

The experiment was conducted in three steps.

### 3.1. Development, Optimization, and Validation of Analytical Method

Given the absence of a pharmacopoeia monograph, an analytical method was developed, optimized, and validated in an attempt to search for degradation products by means of high performance liquid chromatography (HPLC), together with the UV/DAD detector.

The chromatographic studies using HPLC/UV-DAD (Waters Equipment e2695) were performed according to the lovastatin monograph [10,12], and the chromatographic conditions used were: C18 column (ODS, 250 × 4 mm, 5 μm, SunFire) mobile phase: acetonitrile/phosphoric acid 0.1% v/v (65:35), 1.5 mL/min; injection volume of 10 μL, UV detection λ = 238 nm at 303 K, and samples prepared at a concentration of 40 μg/mL in methanol. The method was validated for parameters of precision, accuracy, linearity, limit of detection, limit of quantitation, specificity, and robustness [13]. In addition, the system suitability parameters were calculated according to the United States Pharmacopoeia for the chromatogram, such as the capacity factor (k’), the peak symmetry (As), the Theoretical plates/column (N) and resolution (Rs) between the peaks found [10].

### 3.2. Search for Degradation Products after Exposure to Stress Conditions

The stress conditions (intrinsic stability) of atorvastatin were systematically investigated after 4 h of exposure under distinct conditions: (i) dry heat at 378 K, (ii) reflux over steam bath in water, (iii) in NaOH 1 M, (iv) in HCl 1 M, (v) in an aqueous solution of H_2_O_2_ 3%, and (vi) UV light (254 nm). The HPLC/UV-DAD method used in this study was developed, optimized, and validated according to the lovastatin monograph described in the United States Pharmacopeia [10].

### 3.3. Evaluation of Degradation Kinetics under Acid and Basic Conditions

The drug showed degradation under acid and basic conditions. For this reason, a study was proposed to evaluate the kinetics under these conditions.

Forty mg of drug were weighed and placed in each flask with 0.1 M NaOH (30 mL) and HCl 0.1 M (30 mL). Next, methanol (70 mL) was added to each flask. The flasks were placed in a bath at a temperature of 353 K. Samples were collected after 30, 60, 90, and 120 min and diluted with methanol to a final concentration of 40 μg/mL.

The samples submitted to stress were analyzed by HPLC. Kinetic studies were conducted under acid and basic conditions, according to the model adjustments reported in the literature [14]. Given the analytical results, the order of the degradation reactions was established according to the models of zero, first, and second orders, calculated by applying the linear correlation coefficient (r).

## 4. Conclusions

ATV presented a retention time (t_R_) of 3.517, when samples were subjected to stress under acid and basic conditions. The sample exposedto an acidic medium showed partial degradation with the formation of two degradation products, whereas in the sample subjected to basic hydrolysis, degradation could be observed by a reduction the drug’s peak area.

For the degradation kinetics in acid and basic media, it could be observed that, in the acid medium, the best fit was obtained for first order kinetics, whereas for the basic medium, the best fit corresponded to zero order kinetics.

Considering the kinetic results, the rate constant (k) proved to be higher in the acid medium, 1.88 × 10^−2^ s^−1^ (first order), as compared to the basic medium, k = 2.35 × 10^−4^ mol L^−1^ s^−1^ (zero order), demonstrating a lower stability of the drug in acid media.

## Figures and Tables

**Table 1:** Relationship between retention time, area, and concentration of atorvastatin after submission of the stress in acid medium.

Conditions	Retention time (t_R_) (min)	Área (au)	Concentration (mg/mL)
Atorvastatin	3.843	789,324.00	4 × 10^−2^
After 30 min	3.824	64,904.00	3.289093 × 10^−3^
After 60 min	3.824	59,718.00	3.026286 × 10^−3^
After 90 min	3.824	24,468.00	1.239947 × 10^−3^
After 120 min	3.829	13,311.00	6.74552 × 10^−4^

**Table 2:** Kinetic parameters of degradation in acid medium.

Parameters		Orders	
	Zero (C × time)	First (log C × time)	Second (1/log C × time)
r (linear correlation coefficient)	0.959518923	0.961904152	0.93865055
*k* (rate constant)	3.20999 × 10^−5^	1.8820799 × 10^−2^	13.37113094

**Table 3:** Relationship between retention time, area, and concentration of atorvastatin after submission of the stress in basic medium.

Conditions	Retention time (t_R_) (min)	Área (au)	Concentration (mg/mL)
Atorvastatin	3.843	789,324.00	4 × 10^−2^
After 30 min	3.835	586,493.00	2.972128 × 10^−2^
After 60 min	3.832	446,381.00	2.2620926 × 10^−2^
After 90 min	3.827	340,651.00	1.7262923 × 10^−2^
After 120 min	3.830	157,439.00	7.978422 × 10^−3^

**Table 4:** Kinetic parameters of degradation in basic medium.

Parameters		Orders	
	Zero (C × time)	First (log C × time)	Second (1/log C × time)
r (linear correlation coefficient)	0.99448374	0.96033586	0.903743452
*k* (rate constant)	2.35289 × 10^−4^	1.4054805 × 10^−2^	0.962657291
